# Azithromycin to Prevent Pertussis in Household Contacts, Catalonia and Navarre, Spain, 2012–2013

**DOI:** 10.3201/eid2611.181418

**Published:** 2020-11

**Authors:** Josep Alvarez, Pere Godoy, Pedro Plans-Rubio, Neus Camps, Monica Carol, Gloria Carmona, Ruben Solano, Cristina Rius, Sofia Minguell, Irene Barrabeig, Maria R. Sala-Farré, Raquel Rodriguez, Manuel Garcia-Cenoz, Carmen Muñoz-Almagro, Angela Dominguez, Transmission of Pertussis in Households Working Group

**Affiliations:** Agència de Salut Pública de Catalunya, Barcelona, Spain (J. Alvarez, P. Godoy, P. Plans-Rubio, N. Camps, M. Carol, G. Carmona, S. Minguell, I. Barrabeig, M.R. Sala-Farré, R. Rodriguez);; Institut de Recerca Biomédica de Lleida, Leida, Spain (P. Godoy);; Consorcio de Investigación Biomédica en Red de Epidemiología y Salud Pública (CIBERSP), Madrid, Spain (P. Godoy, P. Plans-Rubio, R. Solano, C. Rius, I. Barrabeig, M. García Cenoz, C. Muñoz-Almagro, A. Domínguez);; Agència de Salut Pública de Barcelona, Barcelona (R. Solano, C. Rius);; Instituto de Salud Pública de Navarra, Pamplona, Spain (M. Garcia-Cenoz);; Hospital de Sant Joan de Deu, Barcelona (C. Muñoz-Almagro);; Universitat de Barcelona, Barcelona (A. Dominguez)

**Keywords:** pertussis, chemoprophylaxis, azithromycin, effectiveness, household contacts, bacteria, antimicrobials, Spain, respiratory infections, prevention, Bordetella pertussis

## Abstract

We retrospectively assessed the effectiveness of azithromycin in preventing transmission of pertussis to a patient’s household contacts. We also considered the duration between symptom onset in the primary patient and azithromycin administration. We categorized contacts into 4 groups: those treated within <7 days, 8–14 days, 15–21 days, and >21 days after illness onset in the primary patient. We studied 476 primary index patients and their 1,975 household contacts, of whom 4.5% were later identified as having pertussis. When contacts started chemoprophylaxis within <21 days after the primary patient’s symptom onset, the treatment was 43.9% effective. Chemoprophylaxis started >14 days after primary patient’s symptom onset was less effective. We recommend that contacts of persons with pertussis begin chemoprophylaxis within <14 days after primary patient’s symptom onset.

After decades of decline (*1*,*2*) and despite high vaccination coverage, the incidence of pertussis has increased substantially in Catalonia, Spain (*3*,*4*); Spain (*5*); and other regions and countries with well-established epidemiologic surveillance systems (*6*). Many researchers attribute this pattern to an increasingly waning immunity in persons vaccinated with the acellular vaccines currently used in most countries instead of the whole-cell vaccines used until the late 1990s (*7*–*11*).

The causative agent of pertussis, *Bordatella pertussis*, is mainly spread through household contacts (*12*,*13*). However, guidelines contain few measures to prevent intrahousehold transmission. Most guidelines recommend patient isolation, vaccination of children <7 years of age, and chemoprophylaxis for household members and other frequent contacts (*14*,*15*). Generally, guidelines recommend that household contacts begin chemoprophylaxis with a macrolide within 21 days after symptom onset in the index patient. However, evidence of its effectiveness in preventing transmission is limited (*16*,*17*). In addition, there is a lack of studies on the effectiveness of azithromycin, because studies on chemoprophylaxis for pertussis usually use erythromycin (*18*,*19*).

A study of pertussis patients in Catalonia and Navarre, 2 autonomous communities in Spain, assessed the overall effectiveness of azithromycin in preventing transmission among household contacts (*20*). After adjustment for age, sex, vaccination history, and relationship to the primary patient, chemoprophylaxis had an adjusted effectiveness of 62.1% in this study, consistent with the results of other studies (*21*). However, this study cohort (*20*) included 164 nonprimary index patients (i.e. patients with the first reported case of pertussis in a household, but not the first chronological case) and their 877 contacts, did not consider the duration between symptom onset in the primary patient and start of treatment, and did not exclude co-primary and tertiary patients (who might not have been infected by the primary patient) (*20*). We assessed whether delays in chemoprophylaxis reduce its effectiveness.

## Materials and Methods

The study cohort comprised the household contacts of primary index patients with pertussis detected by the Epidemiologic Surveillance Units (ESU) of Catalonia and Navarre from January 1, 2012, through December 31, 2013. We followed up on the household contacts 28 days after symptom onset in the index patient.

The index patient was the first patient with pertussis reported to the ESU in each household and the primary patient was the first patient with pertussis in each household, regardless of whether or when his or her case had been reported. In most situations, the index and primary patients were the same person; for our study, we excluded instances when the index and primary patients were different persons. The ESU prescribed the postexposure intervention for every index patient and their contacts. Our study included only patients with *B. pertussis* infection confirmed by culture or real-time PCR of nasopharyngeal samples. We categorized household contacts as persons regularly living in the same household or persons in the home for >2 hours during the transmission period (<21 days after symptom onset in the primary patient or >5 days after the patient’s start of treatment).

ESU staff conducted telephone interviews to gather information about each contact’s age, sex, relationship to the index or primary patient, receipt of chemoprophylaxis and start date, vaccination history, and presence of pertussis symptoms (cough lasting >2 weeks, paroxysmal cough, posttussive vomiting, inspiratory stridor, and apnea). Staff collected vaccination statuses and laboratory results (i.e., culture assay, PCR) from the contacts’ medical records and determined a person’s vaccination status using the vaccination records of each autonomous community. We categorized each contact as fully vaccinated (>4 doses of vaccine), incompletely vaccinated (<4 doses), unvaccinated (no dose), incompletely vaccinated because of age (i.e. children <18 months of age who had received recommended doses), and unvaccinated because of age (i.e. children <2 months of age). Because few contacts >18 years of age had vaccination records, we analyzed this variable only in contacts <18 years of age.

At 28 days after symptom onset in the primary patient, we categorized contacts as follows: healthy contact, no clinical symptoms of pertussis; primary patient, the first patient at a specific address (this might differ from the index patient, who had the first reported case); co-primary patient, symptom onset within <6 days of the primary patient; secondary patient, symptom onset within 7–28 days after the primary patient; and tertiary patient, symptom onset within >28 days after the primary patient. Before administering treatment, ESU staff took nasopharyngeal samples of each patient and their contacts with possible pertussis symptoms. We considered symptomatic contacts as patients when we confirmed their diagnosis by culture or real-time PCR or found an epidemiologic link (onset of symptoms ≤28 days later) with a laboratory-confirmed case.

We evaluated the characteristics of persons who did or did not receive chemoprophylaxis using χ^2^ (for categorical variables) and Student *t*-test (for continuous variables). We then studied the effectiveness of chemoprophylaxis in preventing pertussis in persons classified as healthy contacts or secondary patients after 28 days of follow-up. We excluded co-primary and tertiary patients from the analysis because they might not have been infected by primary patients. 

We calculated the effectiveness of azithromycin for 5 days using the formula effectiveness = (1 − relative risk) × 100. We considered effectiveness according to the duration between symptom onset in the primary patient and start of chemoprophylaxis. We classified this duration into 4 categories: 1–7 days, 8–14 days, 15–21 days, and >21 days after illness onset in the primary patient.

We used unconditional logistic regression to estimate effectiveness adjusted by vaccination status. We also assessed effectiveness according to the age of contacts (<1 year, 1 year, 2–3 years, 4–6 years, 7–10 years, 11–18 years, 19–40 years, and >40 years of age), degree of relationship (cohabitants vs. persons in the home >2 hours), and type of relationship with the primary index patient (mother, father, sibling, grandparent, spouse, child, and other). We analyzed the data using SPSS Statistics 18.0 (IBM, https://www.ibm.com), and Epi Info (Centers for Disease Control and Prevention, https://www.cdc.gov). 

The Ethics Committee of the Hospital Sant Joan de Deu approved the study (code: PIC-79–11). All contacts and family members gave informed written consent to participate.

## Results

From January 1, 2012, through December 31, 2013, the ESU detected 688 cases of pertussis, of which 524 (76.2%) were primary index cases. Of these, 476 (90.8%) case-patients had reported data on the administration and outcome of chemoprophylaxis for 2,051 contacts. We excluded 76 contacts because they were co-primary patients (65 persons) or tertiary patients (11 persons). Therefore, our final study consisted of 1,975 household contacts of 476 primary index patients (hereafter primary patients).

Of the 1,975 contacts we analyzed, 53.5% were female. The mean age was 33.9 (SD ± 20.5) years; 2.2% of contacts were <1 year of age (the most vulnerable group), 34.7% were 19–40 years of age, and 35.4% were >40 years of age. A total of 76.5% of contacts lived with the primary patient; 23.4% of contacts were mothers, 21.5% were fathers, and 19.6% were siblings of the primary patient (Table 1). Most of the 591 contacts <18 years of age were completely vaccinated (65.7% had received >4 doses and 6.1% were completely vaccinated in accordance with recommendations for their age).

**Table 1:** Characteristics of household contacts of primary patients with pertussis, Catalonia and Navarre, Spain, 2012–2013

Characteristic	Contacts*	Received chemoprophylaxis*		p value†
		Yes	No	
Total	1,975 (100)	1,720 (87.1)	255 (12.9)	
Sex				
M	919 (46.5)	797 (46.3)	122 (47.8)	0.64
F	1,056 (53.5)	923 (53.7)	133 (52.2)	
Age, y				
<1	44 (2.2)	41 (2.4)	3 (1.2)	0.67
1	33 (1.7)	33 (1.9)	0 (0.0)	
2–3	79 (4.0)	67 (3.9)	12 (4.7)	
4–6	132 (6.7)	120 (7.0)	12 (4.7)	
7–10	154 (7.8)	135 (7.8)	19 (7.5)	
11–18	149 (7.5)	131 (7.6)	18 (7.1)	
19–40	685 (34.7)	595 (34.6)	90 (35.3)	
>40	699 (35.4)	598 (34.8)	101 (39.6)	
Mean age, y (± SD)	33.9 (20.5)	33.4 (20.5)	37.3 (20.6)	0.005‡
Median age, y	36	36	39	
Type of household contact				
Household cohabitant	1,511 (76.5)	1,311 (76.2)	200 (78.4)	0.44
Other >2 h	464 (23.5)	409 (23.8)	55 (21.6)	
Relationship to primary patient				
Mother	463 (23.4)	400 (23.3)	63 (24.7)	0.69
Father	424 (21.5)	366 (21.3)	58 (22.7)	
Sibling	388 (19.6)	352 (20.5)	36 (14.1)	
Grandparent	281 (14.2)	248 (14.4)	33 (12.9)	
Child	19 (1.0)	15 (0.9)	4 (1.6)	
Spouse	26 (1.3)	20 (1.2)	6 (2.4)	
Other	374 (18.9)	319 (18.5)	55 (21.6)	
Vaccination status <18 y	591	527	64	
Fully vaccinated (>4 doses)	388 (65.7)	349 (66.2)	39 (60.9)	0.36
Incomplete for age	36 (6.1)	35 (6.6)	1 (1.6)	
Incomplete	16 (2.7)	15 (2.8)	1 (1.6)	
Not vaccinated	24 (4.1)	21 (4.0)	3 (4.7)	
Too young for vaccination	5 (0.8)	4 (0.8)	1 (1.6)	
Not stated	122 (20.6)	103 (19.5)	19 (29.7)	
Chemoprophylaxis initiation, d§				
1–7	309 (15.6)	309 (18.0)	0	
8–14	544 (27.5)	544 (31.6)	0	
15–21	413 (20.9)	413 (24.0)	0	
>21	393 (19.9)	393 (22.8)	0	
Unknown	61 (3.1)	61 (3.5)	0	
No chemoprophylaxis	255 (12.9)	255 (14.8)	0	
Type of contact				
Healthy contact	1,886 (95.5)	1,645 (95.6)	241 (94.5)	0.44
Secondary case	89 (4.5)	75 (4.4)	14 (5.5)	

*Values are no. (%) except as indicated. †p value for χ^2^ test. ‡p value for Student *t*-test. §Days after symptom onset of the primary patient.

Of the 1,720 (87.1%) contacts who received chemoprophylaxis, 1,266 (73.6%) were treated within <21 days after symptom onset of the primary patient: 309 (18%) were treated within <7 days, 544 (31.6%) within 8–14 days, 413 (24%) within 15–21 days, and 393 (22.8%) in >21 days. At 28 days after symptom onset in the primary patient, pertussis had developed in 4.5% of contacts, including 1% of those who had received chemoprophylaxis <7 days and 7.6% of those who received it >21 days after symptom onset in the primary patient (Figure). The 1,720 (87.1%) contacts who received and 255 (12.9%) who did not receive chemoprophylaxis differed significantly only by mean age (33.4 vs. 37.3 years) and being a sibling of the primary patient (20.5% vs. 14.1%) (Table 1).

**Figure:**
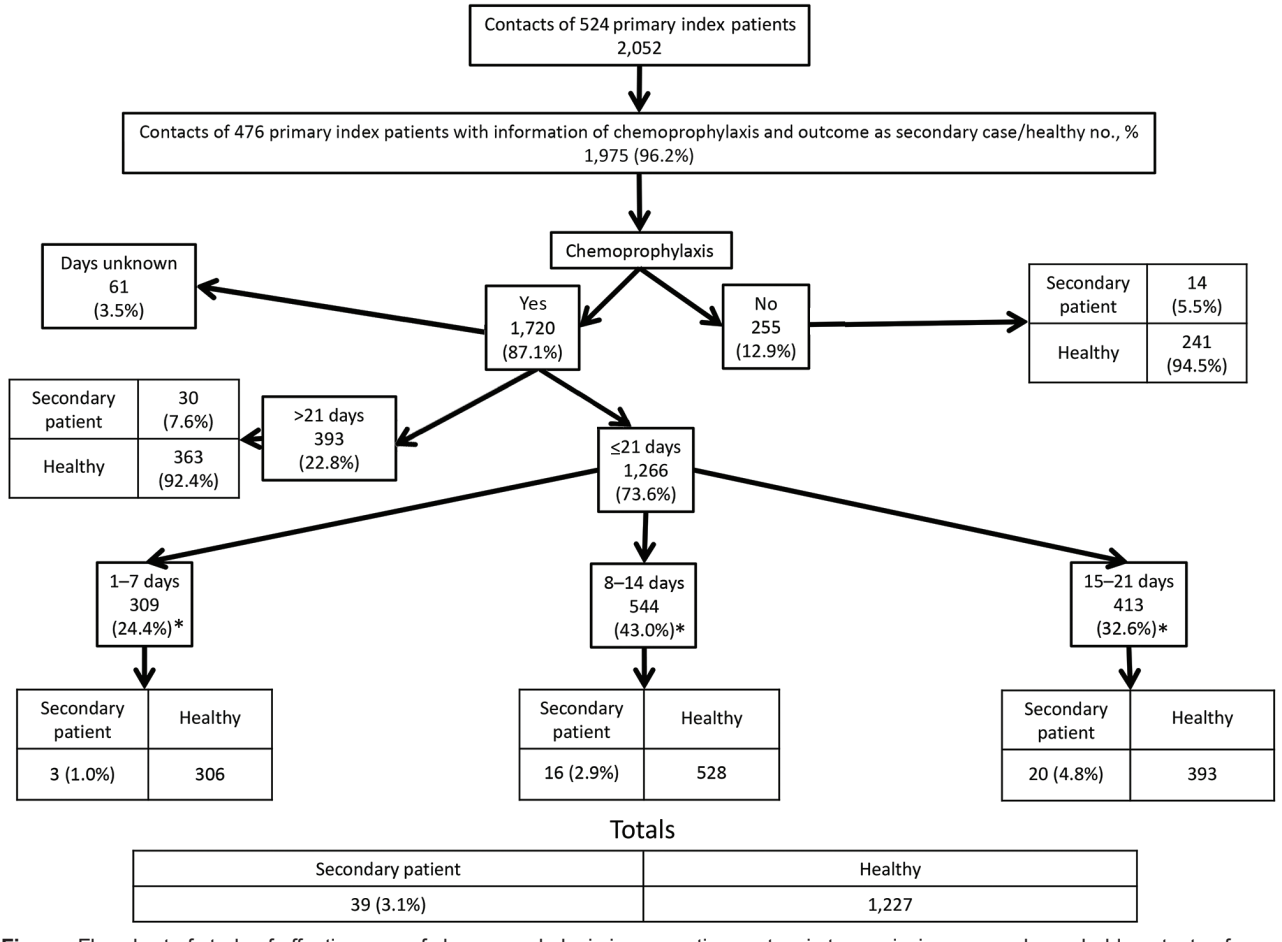
Flowchart of study of effectiveness of chemoprophylaxis in preventing pertussis transmission among household contacts of primary index patients, Catalonia and Navarre, Spain, 2012–2013. *Pooled data.

Chemoprophylaxis was 43.9% (95% CI −1.8% to 69.1%) effective when administered <21 days after symptom onset in the primary patient. Chemoprophylaxis was ineffective (−39.0% [95% CI −157.0% to 25.0%]) when administered after >21 days (Table 2).

**Table 2:** Effectiveness of chemoprophylaxis to prevent pertussis transmission among 1,975 household contacts, Catalonia and Navarre, Spain, 2012–2013

Chemoprophylaxis timing for contacts*	No. contacts	Type of contact		Effectiveness, % (95% CI)
		Healthy contact, no. (%)	Secondary case-patient, no. (%)	
All	1,914	1831 (95.7)	64 (3.3)	
1–21 d	1,266	1,227 (96.9)	39 (3.1)	43.9 (−1.8 to 69.1)
>21 d	393	363 (92.4)	30 (7.6)	−39.0 (−157.0 to 25.0)
No chemoprophylaxis	255	241 (94.5)	14 (5.5)	Reference
Completely vaccinated				
1–21 d	248	233 (94.0)	15 (6.0)	44.1 (−59.5 to 80.4)
No chemoprophylaxis	37	33 (89.2)	4 (10.8)	Reference
Incompletely vaccinated				
1–21 d	50	45 (90.0)	5 (10.0)	50.0 (−248.0, 92.8)
No chemoprophylaxis	5	4 (80.0)	1 (20.0)	Reference

*No. days after symptom onset in primary patient whose contacts received chemoprophylaxis. Includes vaccination status of contacts <18 years of age.

Of contacts <18 years of age, 87.6% (298/340) received chemoprophylaxis, which was 44.1% (95% CI −42.2% to 77.7%) effective. Comparison of contacts <18 years of age who did and did not receive chemoprophylaxis within <21 days showed that chemoprophylaxis was 44.1% (95% CI −59.5% to 80.4%) effective in completely vaccinated persons (≥4 doses) and 50% (95% CI −248.0% to 92.8%) effective in incompletely vaccinated persons. This difference was not significant, although the statistical power was very low (16% for completely vaccinated and 8% for unvaccinated persons) (Table 2).

Overall, in comparison results with contacts who did not receive chemoprophylaxis, the treatment had an effectiveness of 82.3% (95% CI 39.1%–94.9%) for contacts who received it within <7 days, 46.4% (95% CI −8.1% to 73.4%) for those who received it within 8–14 days, and 11.8% (95% CI −71.5% to 54.6%) for those who received it within 15–21 days. When we adjusted the results by vaccination history, we found the reduction over time resembled the declining effectiveness (Table 3).

**Table 3:** Effect of delay in chemoprophylaxis on preventing pertussis transmission among 1,975 household contacts, Catalonia and Navarre, Spain, 2012–2013

Chemoprophylaxis for contacts, d*	No. contacts	Type of contact		Effectiveness, % (95% CI)	Adjusted effectiveness, %† (95% CI)
		Healthy contact, no. (%)	Secondary case-patient, no. (%)		
1–7	309	306 (99.0)	3 (1.0)	82.3 (39.1, 94.9)	89.0 (6.7, 98.7)
8–14	544	528 (97.1)	16 (2.9)	46.4 (−8.1 to 73.4)	37.2 (−114.9 to 75.4)
15–21	413	393 (95.2)	20 (4.8)	11.8 (−71.5 to 54.6)	2.8 (−171.3 to 65.2)
No chemoprophylaxis	255	241 (94.5)	14 (5.5)	Referent	

*No. days after symptom onset in primary patient whose contacts received chemoprophylaxis. †Adjusted by vaccination status.

## Discussion

Most guidelines recommend that contacts take chemoprophylaxis with azithromycin <21 days after symptom onset in the index patient (*14*,*22*–*24*). By only including primary index cases, our study more precisely assessed the effectiveness of chemoprophylaxis in preventing household transmission.

Chemoprophylaxis had an overall effectiveness of 43.9% (95% CI −1.8% to 69.1%), lower than the 62.1% found in the previous study in Catalonia and Navarre (*20*). The effectiveness was highest when given during the first 7 days after symptom onset in the primary patient and fell significantly with increased treatment delays.

Our results reinforce the ineffectiveness (*14*,*24*) (−39.0% [95% CI −157.0% to 25.0%]) of administering chemoprophylaxis >21 days after symptom onset in the primary patient. Chemoprophylaxis also had a low effectiveness when administered after 14 days. Our results indicate that chemoprophylaxis should be started <14 days after symptom onset in the primary patient; however, this recommendation conflicts with the established clinical definition of pertussis, which describes a cough lasting >2 weeks (*22*,*25*). Therefore, we recommend that chemoprophylaxis should start immediately after the ESU is alerted to the possibility of pertussis, without waiting for a laboratory-confirmed diagnosis.

Perhaps because of the small number of persons in each category, effectiveness was not associated with age, vaccination status, degree of home contact, or relationship with the primary patient. However, confounding variables might influence the (lack of) association between vaccination status and chemoprophylaxis effectiveness; for example, vaccine effectiveness might wane in some age groups or be bolstered in persons not cohabiting (i.e., point contact instead of prolonged contact) with the primary patient. The effectiveness of chemoprophylaxis should be more closely investigated in children <1 year of age, in whom pertussis is particularly serious.

Our finding that azithromycin was ineffective when administered >14 days after symptom onset in the primary patient suggests that physicians should not initiate chemoprophylaxis after that time. This strategy might reduce costs, potential adverse effects, and risk for azithromycin resistance (*26*–*28*).

The limited effectiveness of chemoprophylaxis in reducing pertussis transmission highlights the importance of patient isolation until 21 days after symptom onset, or 5 days after treatment initiation (*22*,*24*). Furthermore, communities should strive for high vaccination coverage; physicians should review the vaccination status of contacts; and physicians should regularly update the vaccination schedule, as recommended by some guidelines (*22*). These measures are especially important when a patient with pertussis has contact with children <1 year of age; pregnant women; immunosuppressed persons; and persons with chronic diseases, such as asthma, cystic fibrosis, or congenital heart disease (*6*).

Our study was subject to several limitations. It lacked the statistical power to estimate the effectiveness of chemoprophylaxis in terms of contact age, degree of home contact, and relationship with the primary patient. We used self-reported data on treatment, so we cannot verify whether contacts complied with treatment. We also cannot rule out the possibility that undetected infected persons could have altered transmission dynamics. Finally, confirmatory laboratory testing was not conducted for 40.4% of secondary patients. However, we believe the probability of misclassification is very low.

In conclusion, our results show azithromycin chemoprophylaxis for pertussis had low effectiveness when initiated >14 days after symptom onset in the primary patient. Therefore, public health services should expedite chemoprophylaxis in homes where contacts of suspected patients have risk factors for this disease.
